# Overlapping Leaves Covering Flowers in the Alpine Species *Eriophyton wallichii* (Lamiaceae): Key Driving Factors and Their Potential Impact on Pollination

**DOI:** 10.1371/journal.pone.0164177

**Published:** 2016-10-07

**Authors:** De-Li Peng, Bo Song, Yang Yang, Yang Niu, Hang Sun

**Affiliations:** Key Laboratory for Plant Diversity and Biogeography of East Asia, Kunming Institute of Botany, Chinese Academy of Sciences, Kunming, Yunnan, China; The National Orchid Conservation Center of China; The Orchid Conservation & Research Center of Shenzhen, CHINA

## Abstract

Extrafloral structures are supposed to have evolved to protect flowers from harsh physical environments but might have effects on pollination. Overlapping leaves cover flowers in *Eriophyton wallichii*, an alpine perennial endemic to the Himalaya-Hengduan Mountains. In previous study, it has showed that these extrafloral leaves can protect interior flowers from temperature fluctuations caused by drastic solar radiation fluctuations, but these leaves may also protect interior flowers from rain wash and UVB damage, and we do not know which one is the main function. In this study, we investigated whether rain and UVB protection are the main functions of overlapping leaves covering flowers and their potential impact on pollination. We first measured the intensities of UVB radiation in open air, beneath leaves and corollas, and then examined pollen susceptibility to different intensities of UVB and rain in the laboratory to estimate whether corollas *per se* protect interior pollen from UVB and rain damage. We also carried out pollination treatments and observed pollinator visitation of flowers with and without leaves in the field to assess whether the overlapping leaves covering flowers impair pollinator attraction. Our results showed that (1) water and strong UVB significantly decreased pollen germinability, but corollas *per se* could protect pollen from UVB and rain damage; (2) no autonomous self-pollination and apomixis occurred, and pollinators were essential for the reproduction of *E*. *wallichii*; however, flower coverage by overlapping leaves did not limit pollination. We suggested that rain and UVB protection was not the main function of overlapping leaves covered flowers, given that this protection can be provided by corollas *per se*. Alternatively, this extrafloral structure in *E*. *wallichii* may have evolved in response to extreme high temperatures associated with the strong solar radiation fluctuations. This indicates that, even in alpine plants, extreme high temperature may affect the evolution of plant extrafloral structures.

## Introduction

Heavy rain, strong ultraviolet B radiation (UVB), low temperatures and wide temperature fluctuations are typical characteristics of alpine environments [1]. Among these abiotic agents, rain and UVB can reduce pollen viability [2–7]. Thus, some alpine plants have evolved pollen that is resistant to rain and UVB [7], whereas others rely on protective structures/characteristics of the corolla *per se* [2, 7] or extrafloral structures, such as bent flower stalks and large bracts [3, 8, 9]. In addition to rain and UVB, wide temperature fluctuations and extreme temperatures may also drive the evolution of floral and extrafloral structures [9, 10]. For example, the persistent and upwardly bent calyx of *Anisodus luridus* retains water, thereby ameliorating temperature fluctuations within it [9]. High temperature caused by strong solar radiation reduces pollen viability, so some plants have evolved cooling mechanisms (evaporation and self-shading) in hot climates, such as the tropical zone [11]. In contrast, in arctic or alpine environments, low temperature slows the development of flowers and seeds, thus exerting selective pressure that may be reflected in warming mechanisms, such as flower heliotropism, pubescence and overlapping bracts [12–15].

The evolution of floral/extrafloral structures is shaped by simultaneous selection exerted by both biotic forces, including pollinators and herbivores [16], and their abiotic physical environment [5, 7]. To protect flowers (including pollen), alpine plants have evolved several extrafloral structures to shield flowers from direct exposure to potential abiotic factors [2, 3, 8, 9]. However, they may also reduce the visual display for attracting pollinators. For example, bracts wrap flowers in *Rheum nobile*, protecting pollen from damaging rain, UVB and low temperature [8]. However, the wrapped flowers are less visually attractive for pollinators. Thus, to attract its obligate pollinators, *R*. *nobile* flowers release specific volatiles rather than visual cues [17]. Overall, the benefits conferred by bracts of *R*. *nobile* far exceed the costs, such as increased herbivore attraction or resource consumption [8, 17].

Although abiotic factors have played important roles in the evolution of protective floral and extrafloral structures in alpine plants [18], the key drivers have not yet been fully identified. For example, pendulous flowers may be under simultaneous selection by both rain and UVB [3, 9] and showy translucent bracts may be under simultaneous selection by rain, UVB and low temperature [8]. Thus, it is often difficult to identify the key environmental drivers (if any) of adaptive structures, and there have not been any attempts to do so.

**Fig 1:**
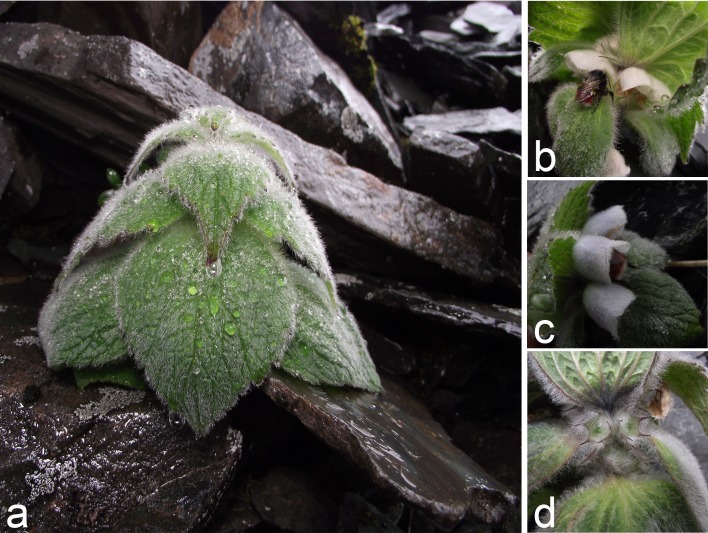
Native habit and gross morphology of *Eriophyton wallichii* and visits by bumblebees: a) the woolly, overlapping leaves covering flowers, b-c) a bumblebee creeping into a corolla to forage for nectar, b-d) the highly pubescent corollas and calyxes.

*Eriophyton wallichii* (Lamiaceae) is a perennial herb endemic to the Himalaya-Hengduan Mountains (HHM). Notable features of this plant include highly pubescent leaves, corollas and calyxes. In particular, woolly and overlapping leaves (extrafloral structures) cover its flowers (Fig 1) and may indeed also protect the flowers from damage by rain and UVB. In a previous study, we showed that the overlapping leaves can protect interior flowers and fruits from temperature fluctuations caused by drastic solar radiation fluctuations [19]. However, it is still unknown whether other abiotic factors (such as rain wash and UVB radiation) also contribute to the retention of this special extrafloral structure.

In the present study, we investigated the relative importance of different abiotic factors in driving the evolution of the overlapping leaves covering flowers, and also assessed whether overlapping leaves make the flowers less attractive for pollinators in *E*. *wallichii*. We addressed the following questions: (1) Which environmental factor best explains overlapping leaves covering flowers? (2) Does *E*. *wallichii* rely on pollinators to reproduce? If so, do the overlapping leaves reduce pollinators’ visitation rates?

## Methods

### Ethics statement

No specific permits were required for the described field studies. The field studies were conducted on public land and did not involve endangered or protected species.

### Study species and sites

*Eriophyton wallichii* is a perennial herb endemic to the HHM subnival belt, and this species is only found in alpine extreme gravelly slopes or screes at altitudes ranging from 4,200 to 5,200 m (De-Li Peng, personal observation). It usually has a large rhizome system but does not seem to propagate vegetatively via rhizomes, i.e. no such reproduction has been observed (De-Li Peng, personal observation). The woolly leaves (extrafloral structures) are circular or sub-circular and overlap flowers (or fruits). One to three subsessile flowers are concealed within each leaf, and each plant typically has 15.55 ± 0.96 flowers (mean ± SE, hereafter; *n* = 67 individuals). Most (or all) of the corollas are covered by leaves (Fig 1). Flowering occurs in early July to late August, and single flowers last 4–6 days [20]. Corollas are white (occasionally pink or light blue), semi-closed, pubescent (Fig 1B) and completely cover the anthers and stigmas. The campanulate calyxes are also pubescent (Fig 1D). Floral maturation is asynchronous, with up to 12 flowers fully open at a time. Flowers are incompletely protandrous but are self-compatible [20]. Each flower produces four ovules with a mean number of 63016 ± 5503 (*n* = 10 flowers) pollen grains, yielding a P/O-ratio of 15754 [20]. Nectar is secreted at the base of the corolla, and each flower produces 2.70 ± 0.44 μL of nectar (*n* = 14 flowers) when they open, with a sugar concentration of 21.91 ± 0.7% (*n* = 34 flowers) [20].

Field experiments were conducted during the summers of 2012 and 2013 at two sites located in the subnival belt of Daxue Shan Mountain in Shangri-la County, Yunnan Province, SW China: one beside Huluhai Lake (99°57′E, 28°31′N, 4,500 m a.s.l.) and the other at Puyong Pass (99°55′E, 28°24′N, 4,620 m a.s.l.). The distance between the two sites was about 70 km (three hours’ drive along a rutted dirt road). Both sites had an open, stony habitat and the vegetation of the two sites was dominated by *E*. *wallichii*, *Phyllophyton complanatum*, *Rhodiola crenulata*, *Fritillaria delavayi* and *Rheum nobile* (De-Li Peng, personal observation). The fields featured a monsoon climate, with 80% of precipitation occurring in the growing season from June to September. The mean annual precipitation (1982–1984) at the nearest meteorological station (99°01′E, 28°23′N, 4,290 m a.s.l.), 95 km from the study sites, was 680–790 mm, with 30 mm in May, 130 mm in June and 500 mm between July and September [21]. The annual average air temperature was -1.0°C, with 2°C in May, 6°C in June, 8°C in July, 7°C in August, 5°C in September and 1°C in October [22]. The instantaneous UVB intensity varied from 70–160 μW cm^-2^ s^-1^ at 14:00 h (strongest solar radiation on sunny days) on 17 and 18 July, 2013 at the Puyong Pass site.

### Effects of rain and UVB on pollen viability

To test the effect of rain on pollen viability, we followed the germination methods of Huang *et al*. [4]. We randomly selected 20 flowers from 20 different individuals (one flower per individual) with newly dehisced anthers from the Puyong Pass population and separated them into two groups of 10. Pollen grains obtained from one group were placed in a 10% (by mass) sucrose solution (judged to be the optimum concentration for pollen germination according to preliminary experiments, De-Li Peng, unpublished data), whereas grains obtained from the second group were placed in distilled water (0% sucrose solution). Pollen grains that had germinated or burst were counted under a light microscope 4 h later [4]. Three replicate sets (to determine the average) of pollen grains from each flower were observed. We used independent-sample *t*-tests to determine differences in rates of both germination and bursting between pollen exposed to water and the sucrose solution. PASW Statistics 18 was used for all statistical analyses reported in this paper [23].

Using UV radiometers supplied by the Photoelectric Instrument Factory of Beijing Normal University, we measured the ground-level UVB intensity and UVB intensities beneath two corollas with and without covering leaves in the same individuals (seven randomly selected plants) at 14:00 h (strongest solar radiation on sunny days) on the 17 and 18 July, 2013 at Puyong Pass population. To test responses of the plant’s pollen grains to UVB, 30 flowers from 30 different individuals (one flower per individual) with newly dehisced anthers were selected randomly and separated into three groups of 10 in the laboratory (at room temperature). The anthers of one group were exposed to UVB (302 nm) at the mean intensity recorded in the open air (110.0 ± 21.1 μW cm^-2^ s^-1^), provided by a UV analyzer (Shanghai Science and Analytical Instruments, Ji-hui, Shanghai). Anthers of another group were exposed to 4.5 ± 0.2 μW cm^-2^ s^-1^ UVB, the mean intensity recorded beneath corollas without covering leaves. The third (control) group was not exposed to UVB. After 4 h, pollen grains from the 10 flowers subjected to each treatment were placed in 10% sucrose solution (at room temperature), and the number of grains from each flower that had germinated after a further 4 h was counted under a light microscope. Three replicate sets of pollen from each flower were used (to determine the average), and each treatment was replicated ten times (*n* = 10 flowers). One-way ANOVA was used to compare pollen germination rates following exposure to the three UVB intensities.

### Breeding system examination

To examine possible contributions of self-pollination to the reproduction of *E*. *wallichii*, and possible adverse effects of pollen limitation on its reproduction, three pollination treatments were carried out on both the Huluhai Lake and Puyong Pass populations (at the field sites) in 2012. To assess apomixis rates, anthers of five randomly selected plants were removed after their flowers had opened but before anther dehiscence, then each selected plant was netted with a nylon mesh bag (10 × 10 threads cm^-2^) to exclude floral visitors. To test if pollinators were required for seed set, intact floral buds of another 10 plants were netted. To test if pollinators were a limiting factor, flowers of a further set of five plants were supplementally pollinated by hand after their flowers had fully opened, using in each case pollen grains from three individuals located at least 10 m away. Thirty naturally pollinated individuals were used as openly pollinated controls. Each manipulated individual was marked with a sign at the base of the stem. All bags used in the treatments were left in place until the end of the experiments. Fruits from all of the individuals used in the tests were collected on the 25^th^ of September, 2012 and then transported to the laboratory to evaluate the fruit set and seed set under each treatment. Three individuals with nylon bags were damaged by yaks from both the Huluhai Lake and Puyong Pass populations, 22 individuals without nylon bags from the Huluhai Lake population were infected by larvae and 24 individuals from the Puyong Pass population were infected by larvae. We removed these damaged individuals. Fruit set was calculated as the number of fruits with seeds divided by the total number of flowers, and seed set as the number of fully developed seeds divided by four (the number of ovules per flower). To test for pollen limitation, we applied an independent-sample *t*-test to determine whether supplemental hand pollination significantly increased frequencies of seeds relative to open pollination.

### Effects of overlapping leaves on floral visitation

Pollinator behavior was observed on sunny days when pollinators were abundant between 10:00 and 17:00 h from 2–6 August, 2012 at the Puyong Pass population. To investigate if the overlapping leaves influenced their visitation rates, we carefully removed all the leaves covering flowers of 55 randomly selected individuals (with 322 flowers). Sixty-five intact individual plants (with 350 flowers) of similar sizes were used as a control group. During 40 (30 min) observation periods (20 h in total), we recorded insects that entered into the corollas of both sets of plants. Visitation rates were standardized as both visits per flower per hour (VFH) and visits per individual per hour (VIH). The difference in pollinator visitation rates (VFH and VIH) between individuals with and without leaves was assessed using independent-sample *t*-tests.

## Results

### Effects of rain and UVB on pollen viability

**Fig 2:**
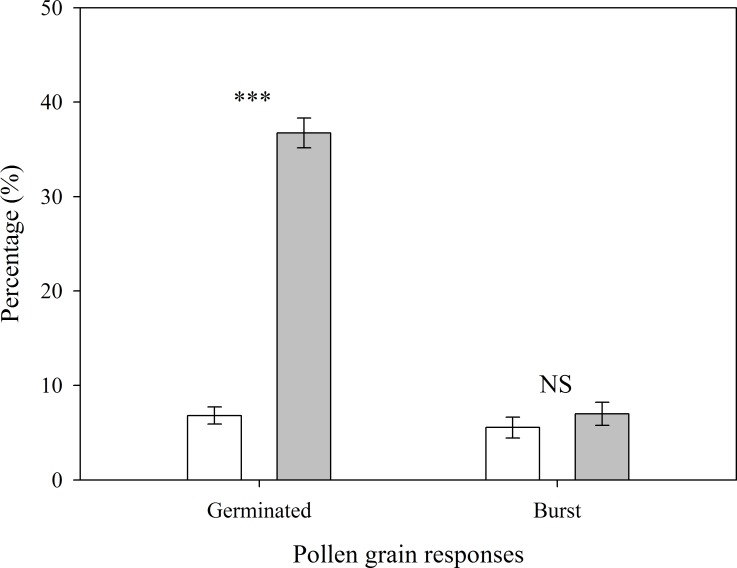
Percentages (means ± SE, *n* = 10) of *Eriophyton wallichii* pollen grains that germinated and burst in 0% (distilled water, open bars) and 10% sucrose solution (filled bars). *** and NS denote a significant difference at the *P* < 0.001 level and no significant difference at the *P* < 0.05 probability level, respectively.

The pollen germination rate was significantly higher in 10% sucrose solution (36.74 ± 1.57%) than in 0% sucrose solution (6.81 ± 0.91%; *t* = 16.484, *P* < 0.001; Fig 2), but their bursting rates did not differ significantly between treatments (*t* = 0.873, *P* = 0.394; Fig 2).

**Fig 3:**
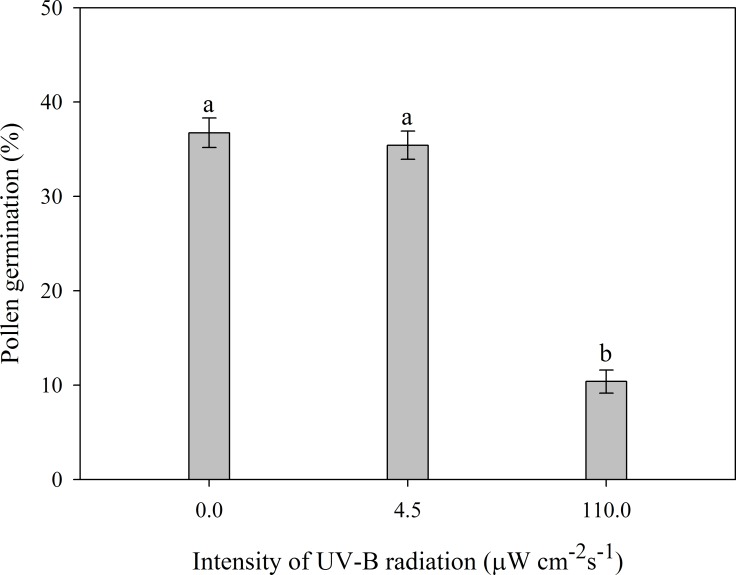
Percentages (means ± SE, *n* = 10) of *Eriophyton wallichii* pollen grains that germinated in 10% sucrose solution after 4 h exposure to indicated intensities of UVB. Different letters denote significant differences at the 0.05 probability level.

Corollas alone reduced the intensity of UVB reaching anthers and stigma by 96%, and the residual UVB (4.5 ± 0.2 μW cm^-2^ s^-1^, *n* = 14 flowers) did not decrease pollen germinability compared to the control treatment (0 μW cm^-2^ s^-1^). However, pollen exposed to strong UVB (110 ± 21.1 μW cm^-2^ s^-1^, *n* = 14 flowers) germinated significantly less frequently (*F*_2, 27_ = 106.874, *P* < 0.001; Fig 3).

### Breeding system examination

Flowers that were only netted, as well as flowers that were netted and emasculated, produced no fruits, suggesting that no autonomous self-pollination and apomixis occurred. Seed set and fruit set were surprisingly high under the open pollination treatment in both populations. Moreover, the seed set of supplementally hand-pollinated flowers was not higher than that of open pollinated flowers (Table 1).

**Table 1:** Fruit set and seed set (mean ± SE) under different pollination treatments. Numbers in parentheses are sample sizes (fruit set: individual number, seed set: flower number).

Treatments	Huluhai lake population		Puyong Pass population	
	Fruit set	Seed set	Fruit set	Seed set
Netting with emasculation	0 (4)	0 (31)	0 (4)	0 (30)
Netting without emasculation	0 (7)	0 (45)	0 (7)	0 (86)
Supplemental pollination	0.77 ± 0.10 (4)	0.64 ± 0.08 (27)	1.00 ± 0.00 (4)	0.83 ± 0.04 (24)
Open pollination	0.86 ± 0.04 (9)	0.67 ± 0.03 (114)	0.92 ± 0.03^*^ (7)	0.76 ± 0.03 (86)

Independent-sample *t*-test was used to determine pollen limitation (supplemental vs. open pollination). Significant difference (at 0.05 level) in fruit set between treatments was only found in population Puyong Pass (marked by “*”).

### Effects of overlapping leaves on floral visitation

**Fig 4:**
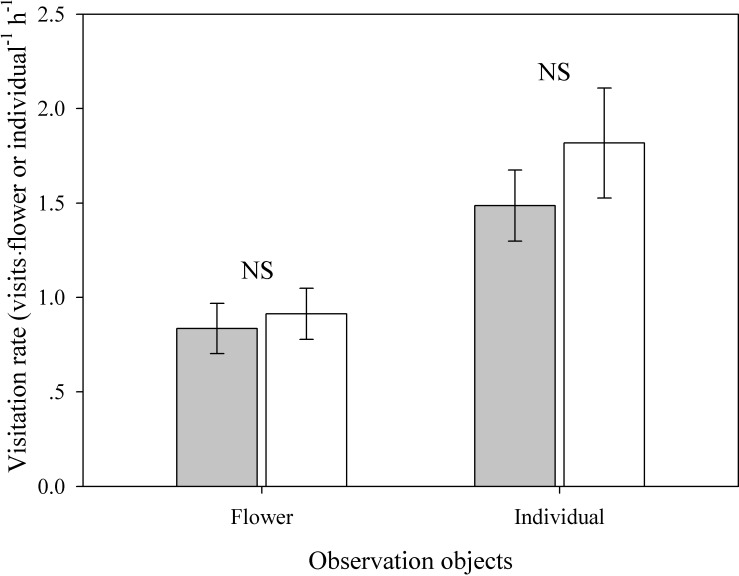
Rates of pollinator visits to plants with removed leaves (open bars, *n* = 19) and intact controls (filled bars, *n* = 21). Data are means ± SE. NS denotes no significant difference at the 0.05 probability level.

The only potential pollinators that visited *E*. *wallichii* during the observation period at the study site were members of a bumblebee species (*Bombus ladakhensis*) species, which pushed their bodies into the corollas to forage for nectar at the base of the corolla tubes. As they crept into the flowers, pollen grains became stuck to their backs and were then available to be transferred to stigmas of the same flower, other flowers of the same individual or other individuals during a continuous visit. The mean visitation rates to the control group (0.84 ± 0.13 VFH and 1.49 ± 0.19 VIH) did not differ significantly from those of the treated group (0.91 ± 0.14 VFH and 1.82 ± 0.29 VIH) at either the individual (*t* = -0.96, *P* = 0.347; Fig 4) or flower level (*t* = -0.41, *P* = 0.690; Fig 4).

## Discussion

### Which environmental factor best explains the evolution of overlapping leaves covering flowers?

*Eriophyton wallichii* begins to bloom in early July during the rainy season in alpine habitats of the HHM, which is characterized by frequent rain and intermittently strong UVB [8, 24, 25]. Wetting by rain and exposure to strong UVB are known to have driven the evolution of some alpine plants’ floral protective structures because they adversely affect pollen viability and germinability [3, 7, 9]. Accordingly, our results showed that exposure to either water or intense UVB significantly reduces germination of *E*. *wallichii* pollen (Figs 2 and 3). Further, although the overlapping leaves covering flowers might provide shelter for pollen grains, the semi-closed corollas also cover the anther and stigma completely (Fig 1B). Pubescence provides water repellency for interior tissues [14, 19], therefore, together with the pubescent structure, the corolla structure may protect pollen from damage caused by rain. Although corollas may not entirely reduce UVB penetration to the interior tissues, the residual intensity of UVB inside the corollas was just 4.1% of the ambient level at the measurement time, corresponding to an intensity of 4.5 μW cm^-2^ s^-1^_,_ which is likely too low to affect pollen viability (Fig 3). Thus, the corollas alone could protect pollen from damage caused by rain and UVB. Therefore, rain and UVB are likely key drivers of the protective corolla’s evolution but probably not the main factors driving the evolution of the overlapping leaves covering the species’ flowers.

In addition to rain and UVB, solar radiation strongly fluctuates during the day in alpine environments. Due to the heating effect of solar radiation coupled with cooling at night (or in cloudy conditions), alpine plants are subject to wide and often rapid temperature variations. Pubescence of leaves and inflorescences is known to be a common plant adaptation to low temperatures [13, 14, 24]. Peng *et al*. [19] have already shown that leaves of *E*. *wallichii* can accumulate heat and the species’ woolly corollas and calyxes (Fig 1D) effectively absorb solar radiation and inhibit convective heat loss [19]. Hence, pubescence of the plant’s floral structures (corolla and calyx) might be an adaptation to low temperatures during the flowering season.

Further, solar radiation can be very intense for short periods in alpine habitats, especially at midday [14, 15, 26]. Hence, flowers of alpine plants reportedly accumulate substantial heat when the sun is out [27]. Furthermore, when the pubescent corollas and calyxes of *E*. *wallichii* are directly exposed to peaks of solar radiation, the flowers and fruits are exposed to high temperatures and wide temperature fluctuations, which reduce pollen germinability and seed production [19]. This potential damage is largely avoided by self-shading provided by the overlapping leaves. In contrast, the effect of overlapping leaves on nighttime radiative cooling is negligible [19]. Thus, comparing to rain, UVB and low temperatures, extreme high temperatures might be the more reasonable factor that responsible for the overlapping leaves covering flowers of *E*. *wallichii*.

### Is visitation influenced by overlapping leaves covering the flowers?

Seed production in the presence of pollinators and lack of seed production in their absence indicate that pollinators are essential for the reproduction of *E*. *wallichii* (Table 1). However, the presence of overlapping leaves does not apparently affect the visitation frequency of pollinators (Fig 4); pollen limitation also did not occur (Table 1). As only a few plants flower at the same time in the alpine scree habitat, pollinators have limited food choices available, whereas flowers of *E*. *wallichii* have rich nectar (2.70 ± 0.43 μL per flower), which could have advantages to attract pollinators. Indeed, the mean visitation rates of *E*. *wallichii* (0.84 VFH, Fig 4) were found to be higher than those of other arctic and alpine plants [28–30]. In addition, *E*. *wallichii* are self-compatible species [20]. When *Bombus* species creep into the corolla to get the nectar, their backs make contact with the anthers and stigma, which results in self-pollination because dichogamy is not complete. After the bumblebees have visited one flower in a plant, they are likely to move to other flowers in the same plant aided by the leaves (De-Li Peng, personal observation). Such foraging behavior can result in considerable self-pollination, enhancing pollination success. Thus, coverage of the flowers by overlapping leaves does not seem to cause a fitness cost.

In summary, the objectives of this study were to test whether UVB and rain protection are responsible for the evolution of the overlapping leaves covering flowers and their potential impact on pollination in *E*. *wallichii*. Our results indicate that rain and UVB protection are not the major function of overlapping leaves covered flowers. Potentially damaging high temperatures during strong solar radiation fluctuations might be responsible for the evolution of the overlapping leaves covering the plant’s flowers as they provide important protection for the reproductive structures. Moreover, these overlapping leaves clearly do not have a negative effect for attracting pollinators. Among the abiotic agents, in addition to UVB, rain and low temperatures, extreme high temperatures caused by large solar radiation fluctuations may have affected the evolution of plant structures in alpine plants.
